# Inhibition of protein kinase CK2 with the clinical-grade small ATP-competitive compound CX-4945 or by RNA interference unveils its role in acute myeloid leukemia cell survival, p53-dependent apoptosis and daunorubicin-induced cytotoxicity

**DOI:** 10.1186/1756-8722-6-78

**Published:** 2013-10-12

**Authors:** Laura Quotti Tubi, Carmela Gurrieri, Alessandra Brancalion, Laura Bonaldi, Roberta Bertorelle, Sabrina Manni, Laura Pavan, Federica Lessi, Renato Zambello, Livio Trentin, Fausto Adami, Maria Ruzzene, Lorenzo A Pinna, Gianpietro Semenzato, Francesco Piazza

**Affiliations:** 1 Department of Medicine, Hematology-Clinical Immunology Branch, and Venetian Institute of Molecular Medicine, Hematological Malignancies Unit, University of Padova School of Medicine, Padova, Italy; 2 Department of Biological Chemistry, University of Padova School of Medicine, Padova, Italy; 3 Department of Oncology and Surgical Sciences, Immunology and Molecular Oncology Unit, Istituto Oncologico Veneto (IOV), Padova, Italy

**Keywords:** Acute myeloid leukemia, Kinase inhibitors, Protein kinase CK2, CX-4945, p53, Daunorubicin, STAT3, Apoptosis

## Abstract

**Background:**

The involvement of protein kinase CK2 in sustaining cancer cell survival could have implications also in the resistance to conventional and unconventional therapies. Moreover, CK2 role in blood tumors is rapidly emerging and this kinase has been recognized as a potential therapeutic target. Phase I clinical trials with the oral small ATP-competitive CK2 inhibitor CX-4945 are currently ongoing in solid tumors and multiple myeloma.

**Methods:**

We have analyzed the expression of CK2 in acute myeloid leukemia and its function in cell growth and in the response to the chemotherapeutic agent daunorubicin We employed acute myeloid leukemia cell lines and primary blasts from patients grouped according to the European LeukemiaNet risk classification. Cell survival, apoptosis and sensitivity to daunorubicin were assessed by different means. p53-dependent CK2-inhibition-induced apoptosis was investigated in p53 wild-type and mutant cells.

**Results:**

CK2α was found highly expressed in the majority of samples across the different acute myeloid leukemia prognostic subgroups as compared to normal CD34^+^ hematopoietic and bone marrow cells. Inhibition of CK2 with CX-4945, K27 or siRNAs caused a p53-dependent acute myeloid leukemia cell apoptosis. CK2 inhibition was associated with a synergistic increase of the cytotoxic effects of daunorubicin. Baseline and daunorubicin-induced STAT3 activation was hampered upon CK2 blockade.

**Conclusions:**

These results suggest that CK2 is over expressed across the different acute myeloid leukemia subsets and acts as an important regulator of acute myeloid leukemia cell survival. CK2 negative regulation of the protein levels of tumor suppressor p53 and activation of the STAT3 anti-apoptotic pathway might antagonize apoptosis and could be involved in acute myeloid leukemia cell resistance to daunorubicin.

## Background

Acute myeloid leukemia (AML) is characterized by the uncontrolled growth of a neoplastic clone of immature myeloid precursors in the bone marrow and in the blood stream. AML encompasses an array of biologically distinct diseases that differ with regard to the pathogenesis, clinical course, response to therapy and prognosis [1]. AML may arise *de novo* or as a secondary cancer in patients previously treated with chemotherapy and/or radiotherapy (therapy-related AML). Malignant clones that are endowed with the capability of escaping spontaneous and drug-induced programmed cell death are selected during the course of the disease. AML - initially responsive to chemotherapy - in a large proportion of cases becomes subsequently refractory to drug-induced apoptosis. Thus, a critical research goal is the identification of the molecular mechanisms accounting for uncontrolled AML cell growth and resistance to apoptosis in order to design novel, molecularly based, targeted therapies [2,3].

Protein kinase CK2 is a ubiquitous serine-threonine kinase involved in a multitude of cellular processes. CK2 is a tetramer enzyme composed most often by two catalytic subunits (α or α’, encoded by separate genes) and two regulatory subunits (β), so that the possible species in the cell are α_2_β_2_ or αα’β_2_[4]. CK2 phosphorylates a large number of substrates with disparate functions [5]. Deletion of CK2α and β in mice is embryonic lethal [6] and knock out of CK2α’ results in globozoospermia and other defects [7]. A remarkable feature of CK2 is the frequent over expression and high enzymatic activity displayed in different types of solid tumors. Indeed, CK2 has been demonstrated to contribute to the malignant phenotype and tumor progression in mouse models as well as in human cancer cells [8]. To this regard, a peculiar property of CK2 is the ability to protect cells from apoptosis [9]. This action is believed to rely on several mechanisms. For instance, CK2 interferes with tumor suppressor PML and PTEN protein stability and function by phosphorylating critical serine residues on these proteins and rendering them less active: in the case of PML through enhanced proteasome-mediated degradation, in the case of PTEN through the stabilization of a less active form of the molecule [10,11]. Moreover, CK2 phosphorylation of anti-apoptotic molecules contributes to protection from apoptosis. CK2 targets Apoptosis Repressor with Caspase Recruiting domain (ARC), shifting the molecule to the mitochondria where it inhibits caspase 8 [12]. Also, CK2 phosphorylation of BID protects it from caspase 8 cleavage and cell death [13]. In addition to this, CK2 positively regulates growth-promoting cascades, such as the PI3K/AKT [14], the NF-κB, the JAK/STAT and the Wnt/β-catenin signaling pathways with the result of strongly directing cell fate towards survival and against programmed cell death [15]. Interestingly, a recently proposed unifying model for CK2 function relies on the regulation of the CDC37/HSP90 chaperone complex through Ser13 phosphorylation on CDC37 [16]. This modification is essential for the chaperoning activity of HSP90 directed towards an array of client protein kinases, many of which are oncogenic. CK2 has also been involved in the cellular DNA damage response, since it was shown that this kinase can regulate both single strand and double strand DNA break repair, by facilitating the XRCC1 function [17] and the UV light response by activating the NF-κB pathway and phosphorylating the high mobility group protein SSRP1 [18,19]. Taken together, the established role played by CK2 in tumorigenesis, could rely on the extraordinary property of this kinase to “addict” cells towards an apoptosis-resistant, proliferation and DNA damage repair-prone-phenotype [20].

However, whereas CK2 expression and activity in a number of solid tumors are more defined, its function in blood cancers is less understood [21]. Kim et al. reported that CK2α is highly expressed in a fraction of cytogenetically normal AML cases and sustains the activation of several pro-survival signaling pathways, since CK2 inhibitors caused AML blast apoptosis [22]. In the present study, we further investigated CK2 expression in a series of AML cases at diagnosis grouped according to the European LeukemiaNet classification [23]. We analyzed the effects of its inhibition in p53 wild-type and mutated AML cell lines and addressed the outcome on anthracycline-driven cytotoxicity. We show that CK2 controls AML cell survival, modulates AML cell sensitivity to daunorubicin and impinge on the p53 and STAT3 survival regulating signaling pathways.

## Results

### Expression levels of CK2 in AML cells

**Figure 1:**
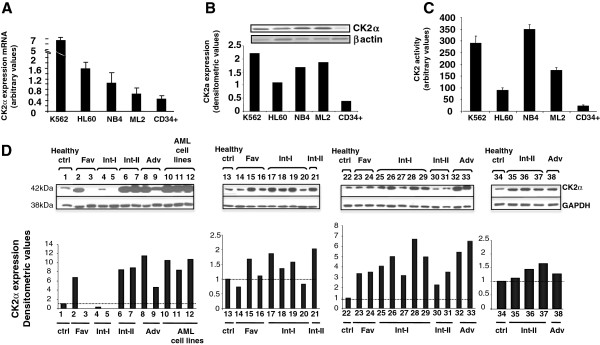
**CK2 expression and activity in AML cells. (A)** Real-time quantitative PCR analysis of CK2α mRNA expression in a panel of AML cell lines (K562, HL-60, NB4, ML2) and in normal CD34^+^ hematopoietic stem cells. **(B)** Top: representative western blot analysis of CK2α protein expression in a panel of AML cell lines (K562, HL-60, NB4, ML2) and in normal CD34^+^ hematopoietic stem cells; bottom: corresponding densitometric analysis. **(C)** In vitro kinase assay measuring CK2 kinase activity against a synthetic peptide using cell lysates from AML cell lines and normal CD34^+^ hematopoietic stem cells. **(D)** Western blot analysis of CK2α expression in normal - or patient derived AML - peripheral blood or bone marrow cells. Thirty-one AML cases were divided according to the European Leukemia Net classification of risk groups in favourable, intermediate-I, intermediate-II and unfavourable. Immunoblots are shown on the upper panel while the corresponding densitometric analysis is shown on the lower panel.

CK2 is over expressed in several solid tumor cells. Kim et al. reported high expression of CK2 also in a subset of AML [22]. In this report, AML cases were grouped according to normal and abnormal karyotype and no differential CK2 expression was observed among the subgroups with abnormal karyotype. Here, we analyzed CK2 expression in AML cell lines and AML cells from patients classified according to the European LeukemiaNet (ELN) classification, which distinguishes different prognostic groups according to cytogenetic alterations and mutations to specific genes [23]. Firstly, quantitative RT-PCR was performed in different cell lines, including K562, NB4, HL-60 and ML2, and normal CD34^+^ hematopoietic cells in order to assess CK2α mRNA levels. As shown in Figure 1A, CK2α mRNA was much higher in AML cell lines as compared to normal CD34^+^ hemopoietic cells. Among the different AML cell lines, K562 was the one displaying the highest CK2α mRNA levels (up to fourteen-fold more as compared to CD34^+^ stem cells); NB4, HL-60 and ML2 showed intermediate (up to seven-fold more as compared to CD34^+^ cells) CK2α levels. CK2α protein levels and CK2 kinase activity were also measured in AML cell lines and CD34^+^ cells (Figure 1B and C). Differently than for the mRNA levels, CK2α protein and activity were found high in K562, ML2 and NB4 but much lower in HL-60 cells. Similar results were obtained when CK2α mRNA and protein levels were compared in AML cells lines and in peripheral blood or bone marrow mononuclear cells (Additional file 1: Figure S1). Next, by Western blot (WB) analysis we analyzed CK2α protein expression across normal peripheral blood or bone marrow cells and primary AML blasts from AML patients. The clinical, biological and genetic features of the samples analyzed are summarized in Table 1. As shown in Figure 1D, CK2α expression was higher in blasts of most of the AML cases, but not all, as compared to normal cells. These results are in accordance with previous observations cited above [22]. Perhaps due to the relatively low number of patients analyzed, we could not detect statistically significant differences among the different ELN AML subgroups upon quantification and densitometric analysis. To note, due to the collection of the samples over different times, four different blots are shown, each created and analyzed by densitometry separately. Thus, the values shown are quite different among the four experiments.

**Table 1:** Features of controls and AML cases analyzed

		**Sample type**	**ELN risk group**	**Karyotype**	**Molecular alterations**	**% CD 34 positive cells**
1	bm	Healthy donor	-	46,XY	-	NA
2	pb	AML patient	Fav	46:xy	NPM1, FLT3 normal, CEBPA mut	60%
3	bm	AML patient	Fav	46,XX,t(15;17)(q22;q11)[24]/46,XX[1] 96% of altered methaphases	PML/RARα	CD34-
4	pb	AML patient	Int-I	46,XY	NPM1 mut (mut A), FLT3 mut	CD34-
5	bm	AML patient	Int-I	46,XY	NPM1and FLT3 normal	82%
6	pb	AML patient	Int-II	47,XY,+19[13]/47,XY,+del(1)(p13)[3]/46,XY[9] 64% of altered methaphases	NPM1 mut (Mut A), FLT3 mut	52%
7	pb	AML patient	Int-II	46,XX,del(1)(p33)[6]/46,XX[1] 86% of altered methaphases	NPM1 mut (mut A), FLT3 normal	CD34-
8	pb	AML patient	Adv	Complex karyotype 100% of altered methaphases	-	78%
9	bm	AML patient	Adv	Complex karyotype 100% of altered methaphases	NPM1and FLT3 normal	89%
10	NB4	AML Cell line	-	-		CD34-
11	ML2	AML Cell line	-	-	PML/RARα	CD34-
12	ME-1	AML Cell line	-	-	-	100%
13	pb	Healthy donor	-	46,XY	-	NA
14	bm	AML patient	Fav	46,XY	NPM1 mut (MUT K) FLT3 normal	27%
15	pb	AML patient	Fav	46,XY		CD34-
16	bm	AML patient	Fav	46,XX	NPM1 Mut (Mut C), FLT3 norm	CD34-
17	pb	AML patient	Int-I	46,XY	NPM1 mut (Mut A),FLT3 mut	48%
18	apheresis	AML patient	Int-I	46,XY	NPM1 mut (Mut A),FLT3 mut	83%
19	pb	AML patient	Int-I	46,XX	NPM1 mut (Mut B), FLT3 mut	10%
20	bm	AML patient	Int-I	46,XY	NPM1 norm, FLT3 mut	CD34-
21	pb	AML patient	Int-II	46,XX	FLT3 mut (+75 ITD), NPM1 normal	CD34-
22	pb	Healthy donor	-	46,XY	-	NA
23	pb	AML patient	Fav	46,XY	NPM1 and FLT3 normal	CD34-
24	pb	AML patient	Fav	46,XX	NPM1and FLT3 normal	CD34-
25	bm	AML patient	Int-I	46,XX	FLT3 mut (+75 ITD), NPM1 norm	22%
26	pb	AMLpatient	Int-I	46,XY	FLT3 mut, NPM1 norm	40%
27	pb	AML patient	Int-I	46,XX	NPM1and FLT3 normal	72%
28	pb	AML patient	Int-I	46,XY	NPM1 mut (mut A), FLT3 mut	CD34-
29	pb	AML patient	Int-I	46,XX	NPM1and FLT3 normal	33%
30	pb	AML patient	Int-II	46,XX,del(1)(p33)[6]/46,XX[1] 86% of altered methaphases	NPM1 mut (mut A), FLT3 normal	CD34-
31	bm	AMLpatient	Int-II	46,XY,t(12;17)(p13;q21)[9]/46,XY[1] 90% of altered methaphases	FLT3 mut (+8 ITD), NPM1 norm	87%
32	pb	AML patient	adv	46,XY,der(8)t(8;?)(p11;?)[20] 100% of altered methaphases	NPM1and FLT3 normal	89%
33	pb	AML patient	adv	Complex karyotype 100% of altered methaphases	NA	78%
34	pb	Healthy donor	-	46,XY	-	NA
35	bm	AML patient	Int-II	46,XY,der(8)t(8;?)(p11;?)[20] 100% of altered methaphases	NPM1and FLT3 normal	76%
36	pb	AML patient	Int-II	NA	NA	90%
37	bm	AML patient	Int-II	NA	NA	95%
38	pb	AML patient	Adv	NA	NA	CD34-

Cases were progressively numbered according to loading order in the immunoblot gels. BM = bone marrow; PB =peripheral blood; FAV = favourable; INT-I = intermediate I; INT-II = intermediate II; ADV = adverse [according to the ELN risk stratification (Rollig et al. 2011, Journal of Clinical Oncology 29;2758)]; NA = not assessed; MUT = mutated.

### Inhibition of CK2 activity causes AML cell apoptosis

**Figure 2:**
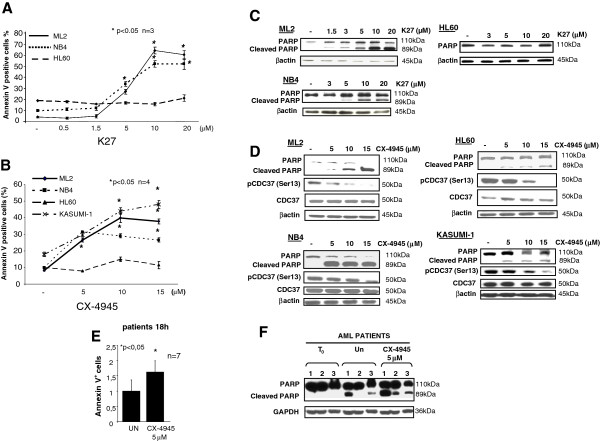
**Pharmacological inhibition of CK2 causes apoptosis of AML cells. (A, B)** Graphs showing the rate of apoptosis assessed by annexin V staining and FACS analysis in a panel of AML cell lines (NB4, ML2 and HL-60, for CX-4945 also Kasumi-1) treated with increasing concentrations of the CK2 inhibitors K27 **(A)** or CX-4945 **(B)**. **(C)** Representative western blot analysis of PARP/cleaved PARP ratio expression in cell lysates from ML2 (top left panels), NB-4 (bottom panels) and HL-60 (top right panels) AML cell lines treated with increasing concentrations of K27. **(D)** Representative western blot analysis of PARP/cleaved PARP ratio and of phospho Ser13 CDC37 and total CDC37 expression in cell lysates from ML2 (top left panels), NB-4 (bottom left panels) and HL-60 (top right panels) and Kasumi-1 (bottom right panels) AML cell lines treated with increasing concentrations of CX-4945. βactin was used to ensure equal protein loading. **(E)** Graph summarizing the rate of apoptosis by annexin V staining and FACS analysis of blasts from AML patients (n = 7; p < 0.05) untreated (un) or treated with 5 μM CX-4945 for 18 hours. **(F)** Western blot analysis of apoptosis as indicated by PARP cleavage in AML blasts protein lysates taken from three AML patients. Proteins were made soon after collection, at time 0 and after 18 hours of culture untreated (Un) or upon exposure to CX-4945 5 μM. Data represent mean ± SD, n = 3. * indicates p < 0.05.

To investigate if CK2 is important for AML cell survival, different AML cell lines were treated with the CK2-specific, ATP-competitive inhibitors, K27 or CX-4945. K27 was characterized in previous studies [24] whereas CX-4945 is a novel orally bioavailable CK2 inhibitor currently under scrutiny in phase I/II clinical trials in USA in different solid tumors and relapsed/refractory multiple myeloma patients [25,26]. After eighteen-hours, cell survival was analyzed by annexin V/propidium iodide (AV/PI) staining and FACS analysis. As a control, cells were treated with the vehicle (DMSO 0.1% in medium). Each AML cell line displayed a different sensitivity to CK2 inhibition. ML2, Kasumi-1 and NB4 cells resulted extremely sensitive to CK2 inhibitors, while HL-60 cells showed a remarkable resistance, being refractory even to high concentrations of the inhibitors (Figure 2A and B). Immunoblot analysis of PARP cleavage or pro-caspase 3 levels confirmed that the treatment of AML cells with the CK2 inhibitors triggered apoptosis in a dose-dependent fashion in ML2 and in NB4, but not in HL-60 cells (Figure 2C and D). The efficacy of CK2 inhibition by the two compounds was confirmed by the decrease levels of CDC37 phosphorylated in Ser13, which is a well-known specific CK2 target [27] (Figure 2D). Most importantly, CX-4945 was highly effective in causing apoptosis of blasts obtained from AML patients, as evidenced by annexin V staining and FACS analysis (*p* < 0.05, n = 7, Figure 2E) and as shown in the representative immunoblot analysis of PARP cleavage (Figure 2F).

### CK2 inhibitors-induced apoptosis is p53-dependent

**Figure 3:**
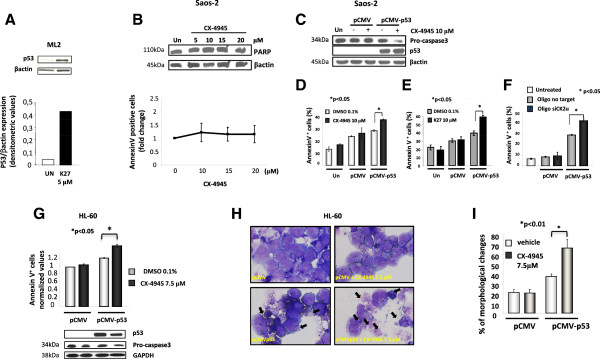
**CK2 controls p53 protein levels in AML cells and p53 is essential for CK2-inhibition triggered apoptosis. (A)** Representative WB analysis on ML2 cells treated with DMSO 0.1% (Un) or 5 μM K27 for 6 hours and probed with an anti-p53 antibody. Graph below: representative densitometric analysis (n = 3; p < 0.05). **(B)** Top: representative WB analysis of total PARP levels in Saos2 osteosarcoma cells treated with CX-4945; bottom: graph summarizing annexin V staining and FACS analysis of Saos2 cells untreated (Un) or treated with increasing concentrations of CX-4945. **(C)** Representative WB of pro-caspase 3 and p53 levels in Saos2 cells untransfected (Un) or transfected with pCMV empty-plasmid (pCMV) or pCMV-p53 expressing plasmid and treated either with DMSO 0.1% (-) or with 10 μM CX-4945. **(D-F)** Graphs summarizing the annexin V staining/FACS analysis of Saos2 cells untransfected (Un) or transfected with pCMV empty (pCMV) or pCMV-p53 plasmid and treated either with DMSO 0.1% or with 15 μM CX-4945 **(D)** or with DMSO 0.1% or with 10 μM K27 **(E)** or re-transfected with scrambled or CK2-directed siRNAs **(F)**. Data represent mean ± SD, n = 3. * indicates p < 0.05. **(G)** Top: graph summarizing the data of annexin V/FACS analysis on HL-60 AML cells transfected with pCMV empty (pCMV) or pCMV-p53 plasmid and treated either with DMSO 0.1% or with 5 μM CX-4945; bottom: representative WB of p53 and pro-caspase 3 protein levels. **(H)** Microscope analysis of Wright-Giemsa stained HL-60 cells transfected with pCMV empty vector or pCMV-p53 and treated either with vehicle (DMSO 0.1%) or CX-4945 7.5 μM. **(I)** Quantification of morphological changes (shrinkage, nuclear picnosis, blebbing or apoptotic bodies) observed in the conditions as in **(H)**. In all the experiments, either βactin or GAPDH levels were determined to ensure equal protein loading.

The observation that HL-60 cells were refractory to the CK2 blockade-induced apoptosis prompted us to test whether this process could be dependent on an intact p53 tumor suppressor function. In fact, HL-60 are p53 null cells, due to gene deletion [28] and this suggests that the AML cell apoptosis upon CK2 inhibition could rely on p53. We thus analyzed p53 levels in wild-type p53-expressing ML2 cells after 6 hours of treatment with 5 μM K27. Upon CK2 inhibition, p53 levels markedly increase as compared to vehicle-treated control cells, indicating that p53 expression and/or stability are negatively regulated by CK2 (Figure 3A). To further support our hypothesis that CK2 inhibition causes apoptosis through p53, we made use of another p53-null cell model system, the human osteosarcoma cell line Saos2 [29]. Saos2 cells were treated with increasing concentration of CX-4945 (ranging from 5 to 20 μM) and cell viability and apoptosis were assessed by immunoblot analysis of PARP cleavage or annexin V staining and FACS analysis. We confirmed that also Saos2 were resistant to CK2-inhibition induced apoptosis, since neither significant PARP cleavage nor annexin V staining could be detected (Figure 3B). Moreover, Saos2 cells transfected with a p53 wild-type expressing vector, but not cells transfected with an empty vector, displayed a significantly increased sensitivity to apoptosis induced by eighteen-hour treatment with either CX-4945 10 μM, as shown by pro-caspase 3 reduction and annexin V staining (Figure 3C and D) or K27 10 μM (*p* < 0.05, n = 3) (Figure 3E) or by siRNA-mediated CK2α down-regulation (Figure 3F). Importantly, a similar induction of apoptosis was obtained in HL-60 AML cells transfected with a p53 expressing vector and treated with CX-4945, as judged by annexin V staining and FACS analysis and immunoblot analysis of p53 (which confirmed the good transfection efficiency) and of pro-caspase 3 levels (Figure 3G; n = 3, p < 0.05). In these set of experiments we also determined the transfection efficiency by employing a pCMV-GFP vector (data not shown). Furthermore, the morphological examination of Wright-Giemsa stained cytological preparations (Figure 3H) and scoring of suffering/apoptotic cells (Figure 3I) also revealed that HL-60 cells’ sensitivity to CX-4945 could be recovered upon over expression of p53. Due to transfection-related toxicity, the basal amount of apoptosis was higher also in mock (pCMV-vector) transfected as compared to untransfected Saos2 and HL-60 cells.

### AML cells show increased sensitivity to daunorubicin upon CK2 inhibition

CK2 inhibition has recently been proposed as a therapeutic strategy to enhance the cytotoxicity of chemotherapeutics [25]. Thus, we sought to investigate whether AML cells would display an increased susceptibility to the cytotoxic effect of the chemoterapeutic drug daunorubicin, a mainstay drug in AML, in conditions of CK2 inhibition.

**Figure 4:**
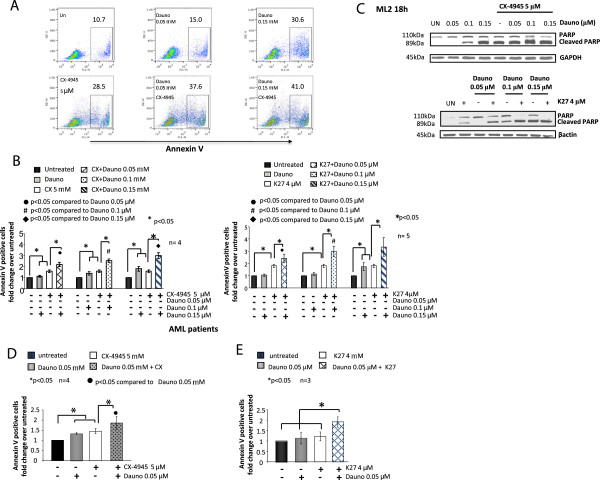
**CK2 inhibition empowers doxorubicin-induced apoptosis of AML cells. (A)** Representative dot plot graph depicting the rate of apoptosis assessed by annexin V staining and FACS analysis of ML2 cells treated with two increasing concentrations of daunorubicin (0.05 and 0.15 μM) without or with a fixed dose of CX-4945 (5 μM). **(B)** Graphs showing ML2 AML cell apoptosis assessed by annexin V staining and FACS analysis in the presence of increasing concentrations of daunorubicin (0.05-0.15 μM) and CX-4945 (leftmost) or K27 (rightmost) kept at the fixed concentrations of 5 μM and 4 μM, respectively. **(C)** Representative immunoblot analysis of PARP cleavage in ML2 cells untreated or treated with increasing concentrations of daunorubicin (0.05-0.15 μM) without or with a fixed subapoptotic dose of CX-4945 (5 μM, top panels) or K27 (4 μM, bottom panels). **(D, E)** graphs summarizing data on the rate of apoptosis as assessed by annexin V staining and FACS analysis of freshly isolated AML blasts treated in the presence of daunorubicin (0.05 μM), CX-4945 5 μM or both **(D)** or daunorubicin (0.05 μM), K27 4 μM or both **(E)**. In all the experiments data represent mean ± SD, n = 3-4. * indicates p < 0.05. β-actin and GAPDH were used as a protein load control in the immunoblot experiments.

To this aim, ML2 cells were subjected to a combination treatment with CX-4945 or K27 at fixed poorly toxic concentrations (5 μM and 4 μM, respectively) and increasing doses (0.05-0.10-0.15 μM) of daunorubicin. Annexin staining and FACS analysis revealed that AML cell sensitivity to daunorubicin was significantly increased by CK2 inhibition either with CX-4945 or with K27 at all the concentrations of the drug tested (p < 0.05, n = 4 or 5) (Figure 4A, representative dot plot of a FACS analysis of CX-4945 effects and Figure 4B, graph summarizing the results from the different experiments with the two inhibitors). Also, immunoblot analysis of PARP cleavage confirmed the cooperative pro-apoptotic effect of CX-4945 or K27 and daunorubicin (Figure 4C). Most importantly, we confirmed the same cooperation between daunorubicin and CK2 inhibitors also on AML blasts isolated from patients (n = 3 or 4), as shown in Figure 4D for CX-4945 and Figure 4E for K27.

### CK2 silencing augments AML cell sensitivity to daunorubicin

**Figure 5:**
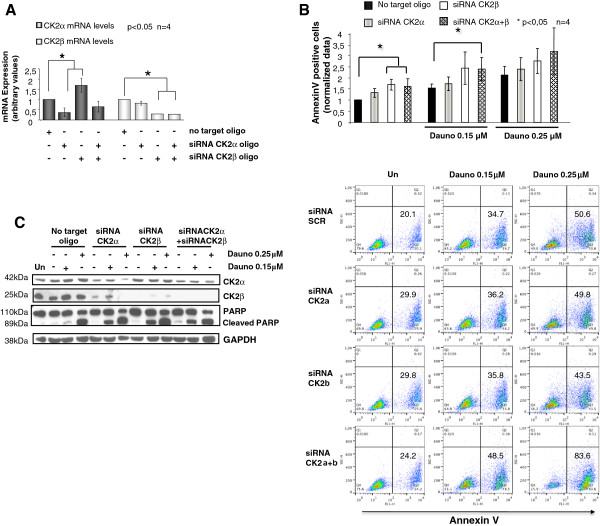
**CK2α and CK2β silencing potentiates daunorubicin-induced AML cell apoptosis. (A)** Graph summarizing the results of real time quantitative PCR analysis of CK2α and CK2β mRNA in ML-2 cells transfected with no target scrambled oligos, CK2α or CK2β-directed siRNAs. **(B)** Histogram graph (top) and dot plot graphs (bottom) summarizing annexin V and FACS analysis of the rate of apoptosis of ML-2 cells upon transfection with no target scrambled, CK2α or CK2β-directed siRNAs without or with exposure to daunorubicin 0.15 μM or 0.25 μM. **(C)** Representative immunoblot analysis of PARP cleavage and CK2α and CK2β protein levels of ML-2 cells transfected with no target scrambled, CK2α or CK2β-directed siRNAs without or with exposure to daunorubicin 0.15 μM or 0.25 μM. In the indicated experiments data represent mean ± SD, n = 4. * indicates p < 0.05.

Lastly, the results obtained with CK2 inhibitors were validated upon silencing of CK2 by mean of RNA interference. A significant down regulation of CK2α and CK2β mRNA could be achieved in ML2 cells transfected with CK2α or CK2β-directed siRNAs and no effects was produced by scrambled oligos (Figure 5A). Interestingly, CK2α mRNA appeared to be up regulated after CK2β silencing. Annexin V staining assay and immunoblot analysis of PARP cleavage demonstrated that silencing of CK2 caused a moderate, though significant, amount of apoptosis of ML2 cells. Most importantly, daunorubicin-induced ML2 cell apoptosis was actually not much enhanced upon silencing of CK2α or CK2β, but it was remarkably boosted upon silencing of both the CK2 subunits (Figure 5B and C; n = 4, p < 0.05).

### Synergic anti-proliferative effect between CK2 inhibitors and daunorubicin on AML cells

**Figure 6:**
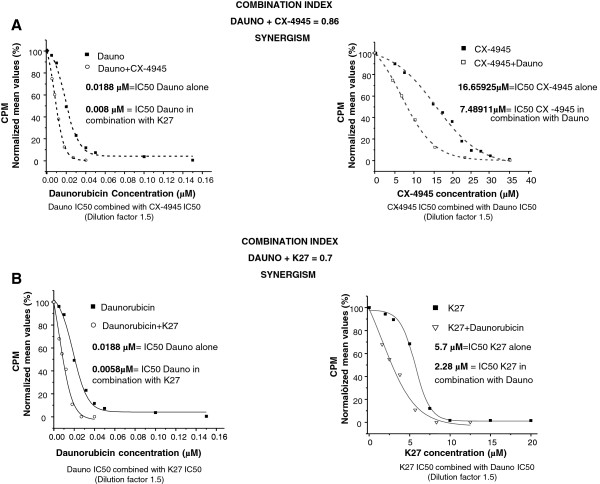
**Calculation of combination indexes indicates a synergistic cell growth arrest upon treatment of the AML cell line ML-2 with the association of daunorubicin and CK2 inhibitors. (A)** Synergistic effect of CX-4945 and daunorubicin on ML-2 cell proliferation. Left graph: dose–response of ML-2 cells incubated for 48 hours with increasing concentrations of daunorubicin (black square) or daunorubicin plus a fixed dose (5 μM) of CX-4945 (white circle). Right graph: dose–response of ML-2 cells incubated for 48 hours with increasing concentrations of CX-4945 (black square) or CX-4945 plus a fixed dose (0.05 μM) of daunorubicin (white square). **(B)** Synergistic effect of K27 and daunorubicin on ML-2 cell proliferation. Left graph: dose–response of ML-2 cells incubated for 48 hours with increasing concentrations of daunorubicin (black square) or daunorubicin plus a fixed dose (5 μM) of K27 (white circle). Right graph: dose–response of ML-2 cells incubated for 48 hours with increasing concentrations of K27 (black square) or K27 plus a fixed dose (0.05 μM) of daunorubicin (white triangle). The combination index (CI), obtained according to the formula described in the Material and Methods section, was calculated as to be: 0.86 for the daunorubicin plus CX-4945 combination and 0.7 for the daunorubicin plus K27 combination, indicating a synergic effect. In all the experiments data represent mean ± SD, n = 3. * indicates *p* < 0.01.

To address whether the cooperation in inducing AML cell death between CK2 inhibition and daunorubicin was synergistic, we performed ^3^H-thymidine incorporation assays evaluating the rate of cell proliferation at increasing concentration of daunorubicin (range: 0.01-0.16 μM), CX-4945 (range: 1–40 μM) and K27 (range: 1–20 μM) and the combination of daunorubicin either with CX-4945 or K27. The results were analyzed to obtain the IC_50_ for the three agents and the *constant ratio drug combination assay* was performed, giving the combination indexes (CI) according to the method described in [30]. The results showed that treatment of ML2 AML cells with daunorubicin and CK2 inhibitors was synergic, as judged by the CI well below 1 (0.86 for the combination with CX-4945 and 0.7 for the combination with K27) (Figure 6A and B, respectively).

### CK2 inhibitors down modulate STAT3 activation upon daunorubicin treatment

**Figure 7:**
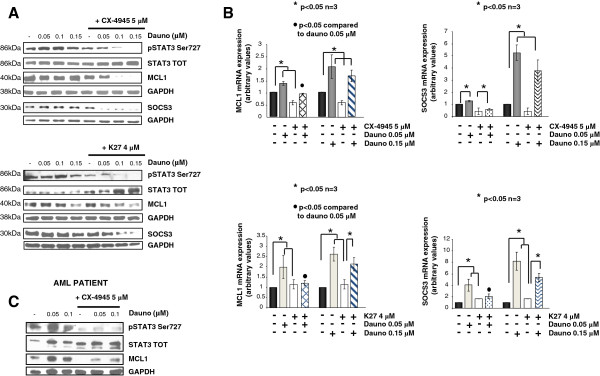
**CK2 inhibition hampers STAT3 activation upon daunorubicin treatment of AML cells. (A)** Representative immunoblot analysis of phospho Ser727 STAT3 (pSTAT3 Ser727), total STAT3, MCL1, SOCS3 protein levels in ML-2 cells untreated, exposed to increasing concentrations of daunorubicin (0.05-0.1-0.15 μM) in the absence or presence of CX-4945 5 μM (top panels) or K27 4 μM (bottom panels). **(B)** Real time quantitative PCR analysis of *MCL1* (lefmost panels) and *SOCS3* (rightmost panels) mRNA in ML-2 cells untreated, exposed to increasing concentrations of daunorubicin (0.05 and 0.15 μM) in the absence or presence of CX-4945 5 μM (top panels) or K27 4 μM (bottom panels). **(C)** Immunoblot analysis of phospho Ser727 STAT3 (pSTAT3 Ser727), total STAT3 and MCL1 in freshly isolated AML blasts from a patient left untreated or exposed to increasing concentrations of daunorubicin (0.05-0.1 μM) in the absence or presence of CX-4945 5 μM. In all the experiments data represent mean ± SD, n = 3. * indicates *p* < 0.05.

Previous work by others’ and our group has demonstrated that CK2 may favour STAT3 activation [31]. STAT3 transcription factors could lend to malignant cells the ability to escape apoptosis induced by a variety of external stimuli, including chemotherapeutic drugs, such as doxorubicin [32-34], and have been described to be important in the pathogenesis of myeloid malignancies [35,36]. Thus, we investigated whether CK2 could regulate STAT3 activation and transcriptional activity, which could account for resistance to daunorubicin in AML cells. As shown in the representative immunoblots in Figure 7A, daunorubicin slightly triggered STAT3 phosphorylation on Ser727. Remarkably, this phosphorylation was nearly completely abrogated by the inhibition of CK2 with either 5 μM CX-4945 (top panels) or 4 μM K27 (bottom panels). The STAT3 pathway may exert its anti-apoptotic function at least in part through the transcriptional up regulation of anti-apoptotic genes, like MCL1. Moreover, a reliable STAT3 target gene is SOCS3, a repressor of cytokine signaling which buffer down the JAK/STAT pathway in a negative feedback loop [37-39]. The immunoblot (Figure 7A) as well as QRT-PCR analysis (Figure 7B) of the expression of these two downstream STAT3 targets demonstrated that both CK2 inhibitors were able to strongly down regulate the transcription and protein expression of MCL1 and SOCS3 (Figure 7A and B) (*p* < 0.05, n = 3). Thus, these results clearly suggest that CK2 inhibition hampers the STAT3-dependent, daunorubicin-elicited anti-apoptotic response in AML cells.

## Discussion

We have shown here that protein kinase CK2 is highly expressed in AML cells regulates AML cell survival and sensitivity to daunorubicin and influences the STAT3 signaling pathway.

CK2 is emerging as a critical cellular serine-threonine kinase that regulates a large array of processes related to cell survival and proliferation [8]. The role of CK2 in sustaining cell in a “non-oncogene addicted” fashion would keep oncogenic pathways constitutively activated [20]. Most of the AML cell lines used in this study displayed increased levels of CK2. Our data confirmed that CK2 up regulation accompanies the transformed phenotype in malignant hematopoietic cells and are in agreement with what has been seen in several types of solid tumor cell lines and tissues [20]. Importantly, CK2 in AML cells sustains survival: in fact, similarly to previous findings from our group in multiple myeloma cells [40] and by others [22] in AML, treatment of AML cells with CK2 specific inhibitors determines the activation of the extrinsic and intrinsic apoptotic pathways, and cell growth arrest (Figure 2). CK2 is known to regulate several mechanisms associated to the extrinsic apoptotic cascades, especially by interfering with the extent of activation of caspase 2 downstream of Fas or TRAIL engagement, thus protecting pro-caspase 8 from cleavage [41-44]. CK2 also regulates the intrinsic apoptotic pathway, even if the mechanisms involved in this regulation are less clear [45]. Remarkably, the cytotoxic effect of the CK2 inhibitors was also evident in freshly isolated AML blasts from patients. Thus, the efficacy of inducing AML cell apoptosis by CK2 inhibitors is similar both in AML cell lines and primary AML cells.

In the present study we have also provided compelling evidence that CK2 inhibition leads to AML apoptosis partly in a p53-dependent fashion. In fact, the p53-mutated HL-60 AML - as well as Saos2 osteosarcoma - cell lines displayed refractoriness to the cytotoxic effect triggered by CX-4945 and K27. Most importantly, restoring a normal p53 function in HL-60 and Saos2 cells rendered these cells prone to apoptosis induced by CK2 inhibitors (Figure 3). The molecular relationships between CK2 and p53 are complex and not fully understood. CK2 can phosphorylate p53 in Ser392 upon UV light exposure and this would increase p53 transcriptional activity [46,47]. However, *in vivo* mouse models of CK2 over-expression showed a synergy with p53 loss in inducing lymphomas [48]. Other studies have also provided evidence that CK2 antagonizes p53 tumor suppressor activity (reviewed in [5]). The results obtained in our study suggest that AML in which p53 function is lost are more resistant to CK2 inhibition-induced cell death and might not need CK2 protein over expression or increased activity, since HL-60 did not display these features (Figure 1B and C). If this turns true also in primary AML blasts from patients remains to be elucidated. We also demonstrated that in p53 wild-type expressing AML cells, CK2 inhibition was accompanied by an accumulation of p53, suggesting that CK2 activity would be important for the regulation of p53 protein turnover in AML cells. Indeed, it is possible that CK2 down regulates p53 protein through the modulation of the COP9 signalosome, a multimolecular complex containing CK2 and other kinases and whose role is to direct a multitude of cellular proteins towards proteaosme mediated degradation [49].

Most importantly, we established that the anti-apoptotic role of CK2 in AML cells is pivotal in protecting AML cells against drug-induced apoptosis. We have shown that daunorubicin, a widely used drug for the therapy of both solid tumors and hematological malignancies, induces AML cell apoptosis at a higher rate in the absence of a functional CK2, suggesting that this kinase might regulate anti-apoptotic signaling pathways involved in daunorubicin-incuced cell death (Figures 4 and 5). The protective effect of CK2 against daunorubicin was evident both in AML cell lines and in AML blasts freshly isolated from AML patients. To note, this effect was synergic (Figure 5). We demonstrated that the STAT3 transcription factor-regulated signaling pathway, which can be elicited upon exposure of tumor cells to chemotherapeutics, was partly activated by daunorubicin in AML cells (Figure 7). Remarkably, we showed that this activation is partially controlled by CK2. Indeed, we provided evidence that CK2 inhibtion was associated with a drop in STAT3 phosphorylation on Ser727 and with a reduction of the transcription of anti-apoptotic STAT3 target genes, like MCL1. Whether CK2 directly phosphorylates STAT3 in AML cells remains to be elucidated. Nevertheless, our data implicate CK2 as a master regulator of the cellular response attempting to antagonize the daunorubicin-induced apoptosis and they show, accordingly to previous studies (reviewed in [15]), that this kinase is essential to maintain STAT3 transcriptional activity.

## Conclusions

The data we present here reinforce the notion that CK2 might control critical signalling cascades in AML, which could not only propel the growth of the bulk of leukemia but also sustain the leukemia stem cell compartment. Indeed, recent work has shown that CK2 inhibition dampens down the PI3K/AKT pathway with a reduction of the activity of downstream effectors such as Bcl-xl and NF-κB and cooperates with PI3K inhibitors in inducing cell death of CD34^+^ CD38^-^ AML leukemia stem cells [50]. Our study provides experimental evidence that AML cell survival relies at least in part on an intact CK2 function. CK2 might protect against chemotherapy-induced cell death through inhibition of p53 and activation of STAT3. Therefore, the pharmacological disruption of CK2 could achieve the goal of restoring p53 function while simultaneously inhibiting STAT3 activity [51] and could be envisioned as a complementary therapeutic strategy in the management of p53 wild-type/STAT3 over expressing AML. This perspective is particularly intriguing in that STAT3 has been demonstrated to regulate leukemia stem cell but not hematopoietic stem cell survival [52]; thus, drugs that target simultaneously STAT3, AKT and NF-κB in AML could produce the effect of eradicating the malignant, but not the normal, stem cell pool.

## Material and methods

### Primary AML blasts, AML cell lines and cultures

Patients were charged to the University of Padova Hospital. Written informed consent was obtained from patients according to the declaration of Helsinki. The project outline and consent procedures and forms were submitted and approved by the Ethic Committee of the Padova University Hospital (protocol number 2612P). Samples from healthy subjects and AML blasts from peripheral blood (PB) and bone marrow (BM) were processed as per standard protocols. From PB blasts were enriched after sheep red blood cells-mediated T-cell depletion. Cases were used when blasts were superior to 80% of total cells. AML cell lines NB-4, ML-2, HL-60 and KASUMI-1 were maintained in RPMI 1640 medium supplemented with L-glutamine, antibiotics (penicillin and streptomycin) and 10% or 20% fetal bovine serum (FBS) according to manufacturer’s datasheet (Gibco Laboratories, Grand Island, NY, USA). Saos2 cells were grown in DMEM with 10% FBS. All cell lines were kept under controlled atmosphere at 37°C in the presence of 5% CO_2_.

Cell cultures were periodically checked for Mycoplasma contamination.

### Chemicals

CK2 inhibitor K27, a TBB-derivative, (2-amino-4,5,6,7-tetrabromo-1H-benzimidazole) was synthesized and kindly provided by Dr Z. Kamizierczuk (Warsaw, Poland); CX-4945 was purchased from Activate Scientific GmbH. Daunorubicin (purchased from Pfizer) was provided by the University of Padua Hospital, Department of Medicine.

### Evaluation of growth and apoptosis

Apoptosis was assessed by fluorescein isothiocyanate (FITC)-Annexin V/Propidium iodide staining (BD Pharmingen) or in separate experiments, by detection of mitochondrial membrane potential using 5,5′,6,6′, tetrachloro 1,1′,3,3′-tetraethylbenzimidazolyl carbocyanin iodide dye (JC-1) (Trevigen, Germany) according to the manufacturer’s instructions. Samples stained with Annexin V/Propidium Iodide or JC-1 were then analyzed by flow cytometry with FACScalibur and CellQuest (Beckton Dickinson) or FlowJo analytic softwares. In order to calculate the combination index, using the Chou-Talalay method [53], the IC_50_ values of different agents were calculated. For this purpose [^3^H]thymidine incorporation assays were performed. AML cells were seeded in 96-well plates (8×10^4^/well) with different concentration of CK2 inhibitors (K27 or CX-4945) or daunorubicin. After 40 hours [^3^H]thymidine was added to the cultures (10 μCi/well) for at least 8 h. The [^3^H]thymidine incorporation was evaluated by scintillation counting by using a β-counter (Microbeta Plus, Wallac). The IC_50_ concentrations of single agents were combined by keeping a fixed ratio for the treatment of AML cells.

### CK2 activity in cell lysates

CK2 activity was measured on the R_3_AD_2_SD_5_ peptide substrate, as previously described [40].

### RNA interference, plasmids and transfection

RNA interference was performed using small interfering RNAs purchased from Dharmacon, USA: ML-2 and HL-60 cells (2 × 10^6^), Saos2 (1 × 10^6^) in log phase of growth were nucleofected with the Amaxa system kit V (using L-029 program for ML-2, X-001 program for HL-60 and Saos2); cells were transfected with 100 pmol siGLO Green scrambled siRNA (as marker of transfection efficiency), control siRNA pool (no targeting pool) or CK2α/CK2β-specific siRNA pool. (CK2α specific target sequences were:GCAUUUAGGUGGAGACUUC; GGAAGUGUGUCUUAGUUAC; GCUGGUCGCUUACAUCACU; AACAUUGUCUGUACAGGUU; CK2β-specific target sequences were: CAACCAGAGUGACCUGAUU; GCAAGGAGACUUUGGUUAC; GCAAUGAAUUCUUCUGUGA; CCAAGUGCAUGGAUGUGUA). Cells were immediately put in pre-warmed RPMI or DMEM media and left in culture for 72 hours. In the daunorubicin treatment experiments, cells were exposed to the chemical after 48 hours from transfection and collected 18 hours later. HL-60 and Saos2 cell lines were also transiently transfected with the empty pCMV plasmid vector or pCMV-p53 wild-type expression vector (kind gift of Dr. P.P. Pandolfi, Harvard University, MA, USA); HL-60 were nucleofected by means of the Amaxa system® using 1 μg of each plasmid, Saos2 were transfected through lipofectamine 2000 (Invitrogen, Carlsbad, CA) using 3 μg of each plasmid and a DNA/lipofectamine ratio of 1:2.5. The over expression of p53 was then evaluated by western blot analysis. After 48 hours from transfection with pCMV o pCMV-p53 vectors cells were treated for 18 hours with CK2 chemical inhibitors or transfected with scrambled or CK2α-specific siRNA oligos.

### Microscopy

HL-60 cells (8 × 10^4^) were spotted on glass slides through citospin and then stained with May-Grünwald/Giemsa method: 3′ in pure May-Grünwald; 3′ in May-Grünwald diluted 1:2 v/v in water, 20′ in Giemsa diluted 20% v/v in water, final wash in water. Samples were analysed by means of Olympus CX-41 microscope at 20× magnification.

### Western blot and antibodies

Whole cell extracts (WCE) were obtained by lysis with 20 mM Tris (pH 7.5), 150 mM NaCl, 2 mM EDTA, 2 mM EGTA supplemented with 0,5% Triton X-100 (Sigma-Aldrich), protease inhibitor cocktail (Sigma-Aldrich), phosphatase inhibitor cocktail (Thermo Scientific), 1 mM phenyl-methyl-sulfonyl fluoride (PMSF; Sigma-Aldrich), 1 μM okadaic acid (Sigma-Aldrich). Twenty to 50 μg of WCE were subjected to SDS-PAGE, transferred to nitrocellulose or PVDF membranes and immunoblotted with the following primary antibodies: CK2α-subunit rabbit antiserum raised against the (376–3919) region of human protein (kindly provided by Dr S. Sarno, University of Padua, Italy); anti-PARP, anti-STAT3 and phospho-Ser727-STAT3, anti-MCL1 (Cell Signaling, Beverly, MA); anti-SOCSs3 and anti-CDC37 (Santa Cruz Biotechnology, Santa Cruz, CA) anti-phospho-Ser13 CDC37 (Abcam); anti-caspase 3 (Calbiochem-Merck Biosciences, bad Soden, Germany); anti-p53 and CK2β (BD Biosciences, USA); βactin (Sigma-Aldrich), GAPDH (Ambion). As secondary antibodies: anti-rabbit IgG HRP-linked antibody (Cell Signaling, Beverly, MA); HRP labeled goat anti-mouse IgG (KPL, Gaithersburg, MD, USA). Detection was performed using ECL (Pierce, Thermo Scientific), Super Signal West Pico Chemiluminescent Substrate (Pierce, Thermo Scientific) or LiteAblot Extend Long Lasting Chemiluminescent Substrate (Euroclone) according to manufacturer’s instruction. Densitometric analysis was conducted using Quantity One Software (Biorad).

### Real-time quantitative PCR

cDNA (50 ng) was subjected to real-time RT-PCR using SYBR Green Reagents (Invitrogen) according to manufacturer’s protocol. The incorporation of SYBR Green Dye into the PCR products was monitored in real-time with ABI PRISM 7000 detection system (Applied Biosystem). Target genes were quantified relative to a reference gene, glyceraldehydes-3-phosphate dehydrogenase (*GAPDH*), which expression was stable in our experimental condition. Primer sequences are the following ones: *CK2α* (F: TCATGAGCACAGAAAGCTACGA) (R: AATGGCTCCTTCCGAAAGATC; *CK2β* (F: CCCATTGGCCTTTCAGACAT) (R: CCGTGTGATGGTGTCTTGATG); *MCL-1*(F:GAAAGTATCACAGACGTTCTCGTAAGG) (R:AACCCATCCCAGCCTCTTTG); *SOCS3* (F: CAGCTCCAAGAGCGAGTACCA) (R: AGAAGCCGCTCTCCTGCAG); *GAPDH* (F: AATGGAAATCCCATCACCATCT; (R: CGCCCCACTTGATTTTGG)*;*

### Statistical analysis

Statistical significance of data was evaluated with the 2-tailed paired Student *t* test or analysis of variance (ANOVA) with post-hoc corrections. Results were considered statistically significant at *p* values below 0.05.

## Competing interests

The authors declare that they have no competing interests.

## Authors’ contributions

LQT, AB, LP, SM, CG and FP performed the research. CG and FP designed the research and the experiments and analyzed the data. LB and RB performed the cytogenetic and molecular diagnosis. GS and FP supervised the research and provided funding. LAP, RZ, LT, FA provided samples and discussed the results. MR performed experiments, contributed to discussion and analyzed the data. FP wrote the manuscript. All authors read and approved the final manuscript.

## Supplementary Material

Additional file 1: Figure S1. CK2 expression and activity in AML cell lines and normal mononuclear cells. (A) Real-time quantitative PCR analysis of CK2α mRNA expression in a panel of AML cell lines (K562, HL-60, NB4, ML2) and in normal peripheral blood (pb) cells. (B) Top: representative western blot analysis of CK2α protein expression in a panel of AML cell lines (K562, HL-60, NB4, ML2) and in normal peripheral blood (pb) or bone marrow (bm) cells; bottom: graph showing the corresponding densitometric analysis. Click here for file

## References

[B1] Lowenberg B Acute myeloid leukemia: the challenge of capturing disease variety Hematology Am Soc Hematol Educ Program 2008 2008 1 11 19074046 10.1182/asheducation-2008.1.1

[B2] Kennedy JA Barabe F Investigating human leukemogenesis: from cell lines to in vivo models of human leukemia Leukemia 2008 22 11 2029 2040 18685615 10.1038/leu.2008.206

[B3] Buzzai M Licht JD New molecular concepts and targets in acute myeloid leukemia Curr Opin Hematol 2008 15 2 82 87 18300752 10.1097/MOH.0b013e3282f3ded0

[B4] Mazzorana M Pinna LA Battistutta R A structural insight into CK2 inhibition Mol Cell Biochem 2008 316 1–2 57 62 18626746 10.1007/s11010-008-9822-5

[B5] Gyenis L Litchfield DW The emerging CK2 interactome: insights into the regulation and functions of CK2 Mol Cell Biochem 2008 316 1–2 5 14 18553055 10.1007/s11010-008-9830-5

[B6] Lou DY Dominguez I Toselli P Landesman-Bollag E O’Brien C Seldin DC The alpha catalytic subunit of protein kinase CK2 is required for mouse embryonic development Mol Cell Biol 2008 28 1 131 139 17954558 10.1128/MCB.01119-07 PMC2223292

[B7] Seldin DC Lou DY Toselli P Landesman-Bollag E Dominguez I Gene targeting of CK2 catalytic subunits Mol Cell Biochem 2008 316 1–2 141 147 18594950 10.1007/s11010-008-9811-8 PMC3696998

[B8] Trembley JH Wang G Unger G Slaton J Ahmed K Protein kinase CK2 in health and disease: CK2: a key player in cancer biology Cell Mol Life Sci 2009 66 11–12 1858 1867 19387548 10.1007/s00018-009-9154-y PMC4385580

[B9] Ahmad KA Wang G Unger G Slaton J Ahmed K Protein kinase CK2–a key suppressor of apoptosis Adv Enzyme Regul 2008 48 179 187 18492491 10.1016/j.advenzreg.2008.04.002 PMC2593134

[B10] Scaglioni PP Yung TM Cai LF Erdjument-Bromage H Kaufman AJ Singh B Teruya-Feldstein J Tempst P Pandolfi PP A CK2-dependent mechanism for degradation of the PML tumor suppressor Cell 2006 126 2 269 283 16873060 10.1016/j.cell.2006.05.041

[B11] Torres J Pulido R The tumor suppressor PTEN is phosphorylated by the protein kinase CK2 at its C terminus: implications for PTEN stability to proteasome-mediated degradation J Biol Chem 2001 276 2 993 998 11035045 10.1074/jbc.M009134200

[B12] Li PF Li J Muller EC Otto A Dietz R von Harsdorf R Phosphorylation by protein kinase CK2: a signaling switch for the caspase-inhibiting protein ARC Mol Cell 2002 10 2 247 258 12191471 10.1016/s1097-2765(02)00600-7

[B13] Olsen BB Petersen J Issinger OG BID, an interaction partner of protein kinase CK2alpha Biol Chem 2006 387 4 441 449 16606343 10.1515/BC.2006.059

[B14] Di Maira G Salvi M Arrigoni G Marin O Sarno S Brustolon F Pinna LA Ruzzene M Protein kinase CK2 phosphorylates and upregulates Akt/PKB Cell Death Differ 2005 12 6 668 677 15818404 10.1038/sj.cdd.4401604

[B15] Dominguez I Sonenshein GE Seldin DC Protein kinase CK2 in health and disease: CK2 and its role in Wnt and NF-kappaB signaling: linking development and cancer Cell Mol Life Sci 2009 66 11–12 1850 1857 19387549 10.1007/s00018-009-9153-z PMC3905806

[B16] Miyata Y Nishida E CK2 controls multiple protein kinases by phosphorylating a kinase-targeting molecular chaperone, Cdc37 Mol Cell Biol 2004 24 9 4065 4074 15082798 10.1128/MCB.24.9.4065-4074.2004 PMC387775

[B17] Loizou JI El-Khamisy SF Zlatanou A Moore DJ Chan DW Qin J Sarno S Meggio F Pinna LA Caldecott KW The protein kinase CK2 facilitates repair of chromosomal DNA single-strand breaks Cell 2004 117 1 17 28 15066279 10.1016/s0092-8674(04)00206-5

[B18] Kato T Jr Delhase M Hoffmann A Karin M CK2 Is a C-terminal IkappaB kinase responsible for NF-kappaB activation during the UV response Mol Cell 2003 12 4 829 839 14580335 10.1016/s1097-2765(03)00358-7

[B19] Li Y Keller DM Scott JD Lu H CK2 phosphorylates SSRP1 and inhibits its DNA-binding activity J Biol Chem 2005 280 12 11869 11875 15659405 10.1074/jbc.M413944200 PMC3923407

[B20] Ruzzene M Pinna LA Addiction to protein kinase CK2: a common denominator of diverse cancer cells? Biochim Biophys Acta 2010 1804 3 499 504 19665589 10.1016/j.bbapap.2009.07.018

[B21] Piazza F Manni S Ruzzene M Pinna LA Gurrieri C Semenzato G Protein kinase CK2 in hematologic malignancies: reliance on a pivotal cell survival regulator by oncogenic signaling pathways Leukemia 2012 26 6 1174 1179 22289987 10.1038/leu.2011.385

[B22] Kim JS Eom JI Cheong JW Choi AJ Lee JK Yang WI Min YH Protein kinase CK2alpha as an unfavorable prognostic marker and novel therapeutic target in acute myeloid leukemia Clinical cancer research: an official journal of the American Association for Cancer Research 2007 13 3 1019 1028 17289898 10.1158/1078-0432.CCR-06-1602

[B23] Dohner H Estey EH Amadori S Appelbaum FR Buchner T Burnett AK Dombret H Fenaux P Grimwade D Larson RA Diagnosis and management of acute myeloid leukemia in adults: recommendations from an international expert panel, on behalf of the European leukemiaNet Blood 2010 115 3 453 474 19880497 10.1182/blood-2009-07-235358

[B24] Sarno S Pinna LA Protein kinase CK2 as a druggable target Mol Biosyst 2008 4 9 889 894 18704226 10.1039/b805534c

[B25] Siddiqui-Jain A Bliesath J Macalino D Omori M Huser N Streiner N Ho CB Anderes K Proffitt C O’Brien SE CK2 inhibitor CX-4945 suppresses DNA repair response triggered by DNA-targeted anticancer drugs and augments efficacy: mechanistic rationale for drug combination therapy Molecular cancer therapeutics 2012 11 4 994 1005 22267551 10.1158/1535-7163.MCT-11-0613

[B26] Pierre F Chua PC O’Brien SE Siddiqui-Jain A Bourbon P Haddach M Michaux J Nagasawa J Schwaebe MK Stefan E Pre-clinical characterization of CX-4945, a potent and selective small molecule inhibitor of CK2 for the treatment of cancer Molecular and cellular biochemistry 2011 356 1–2 37 43 21755459 10.1007/s11010-011-0956-5

[B27] Miyata Y Nishida E CK2 controls multiple protein kinases by phosphorylating a kinase-targeting molecular chaperone, Cdc37 Molecular and cellular biology 2004 24 9 4065 4074 15082798 10.1128/MCB.24.9.4065-4074.2004 PMC387775

[B28] Ju JF Banerjee D Lenz HJ Danenberg KD Schmittgen TC Spears CP Schonthal AH Manno DJ Hochhauser D Bertino JR Restoration of wild-type p53 activity in p53-null HL-60 cells confers multidrug sensitivity Clin Cancer Res 1998 4 5 1315 1322 9607592

[B29] Ryan KM Ernst MK Rice NR Vousden KH Role of NF-kappaB in p53-mediated programmed cell death Nature 2000 404 6780 892 897 10786798 10.1038/35009130

[B30] Manni S Brancalion A Tubi LQ Colpo A Pavan L Cabrelle A Ave E Zaffino F Di Maira G Ruzzene M Protein kinase CK2 protects multiple myeloma cells from ER stress-induced apoptosis and from the cytotoxic effect of HSP90 inhibition through regulation of the unfolded protein response Clinical cancer research: an official journal of the American Association for Cancer Research 2012 18 7 1888 1900 22351691 10.1158/1078-0432.CCR-11-1789

[B31] Piazza F Manni S Semenzato G Novel players in multiple myeloma pathogenesis: role of protein kinases CK2 and GSK3 Leuk Res 2013 37 2 221 227 23174190 10.1016/j.leukres.2012.10.016

[B32] Rebbaa A Chou PM Mirkin BL Factors secreted by human neuroblastoma mediated doxorubicin resistance by activating STAT3 and inhibiting apoptosis Mol Med 2001 7 6 393 400 11474132 PMC1950050

[B33] Alas S Bonavida B Inhibition of constitutive STAT3 activity sensitizes resistant non-Hodgkin’s lymphoma and multiple myeloma to chemotherapeutic drug-mediated apoptosis Clinical cancer research: an official journal of the American Association for Cancer Research 2003 9 1 316 326 12538484

[B34] Gariboldi MB Ravizza R Molteni R Osella D Gabano E Monti E Inhibition of Stat3 increases doxorubicin sensitivity in a human metastatic breast cancer cell line Cancer Lett 2007 258 2 181 188 17920763 10.1016/j.canlet.2007.08.019

[B35] Redell MS Tsimelzon A Hilsenbeck SG Tweardy DJ Conditional overexpression of Stat3alpha in differentiating myeloid cells results in neutrophil expansion and induces a distinct, antiapoptotic and pro-oncogenic gene expression pattern J Leukoc Biol 2007 82 4 975 985 17634277 10.1189/jlb.1206766

[B36] Redell MS Ruiz MJ Alonzo TA Gerbing RB Tweardy DJ Stat3 signaling in acute myeloid leukemia: ligand-dependent and -independent activation and induction of apoptosis by a novel small-molecule Stat3 inhibitor Blood 2011 117 21 5701 5709 21447830 10.1182/blood-2010-04-280123 PMC3110027

[B37] Jourdan M De Vos J Mechti N Klein B Regulation of Bcl-2-family proteins in myeloma cells by three myeloma survival factors: interleukin-6, interferon-alpha and insulin-like growth factor 1 Cell death and differentiation 2000 7 12 1244 1252 11175262 10.1038/sj.cdd.4400758 PMC2423422

[B38] Buettner R Mora LB Jove R Activated STAT signaling in human tumors provides novel molecular targets for therapeutic intervention Clinical cancer research: an official journal of the American Association for Cancer Research 2002 8 4 945 954 11948098

[B39] Ding BB Yu JJ Yu RY Mendez LM Shaknovich R Zhang Y Cattoretti G Ye BH Constitutively activated STAT3 promotes cell proliferation and survival in the activated B-cell subtype of diffuse large B-cell lymphomas Blood 2008 111 3 1515 1523 17951530 10.1182/blood-2007-04-087734 PMC2214773

[B40] Piazza FA Ruzzene M Gurrieri C Montini B Bonanni L Chioetto G Di Maira G Barbon F Cabrelle A Zambello R Multiple myeloma cell survival relies on high activity of protein kinase CK2 Blood 2006 108 5 1698 1707 16684960 10.1182/blood-2005-11-013672

[B41] Ravi R Bedi A Sensitization of tumor cells to Apo2 ligand/TRAIL-induced apoptosis by inhibition of casein kinase II Cancer Res 2002 62 15 4180 4185 12154014

[B42] Izeradjene K Douglas L Delaney A Houghton JA Casein kinase II (CK2) enhances death-inducing signaling complex (DISC) activity in TRAIL-induced apoptosis in human colon carcinoma cell lines Oncogene 2005 24 12 2050 2058 15688023 10.1038/sj.onc.1208397

[B43] Wang G Ahmad KA Ahmed K Role of protein kinase CK2 in the regulation of tumor necrosis factor-related apoptosis inducing ligand-induced apoptosis in prostate cancer cells Cancer Res 2006 66 4 2242 2249 16489027 10.1158/0008-5472.CAN-05-2772

[B44] Llobet D Eritja N Encinas M Llecha N Yeramian A Pallares J Sorolla A Gonzalez-Tallada FJ Matias-Guiu X Dolcet X CK2 controls TRAIL and Fas sensitivity by regulating FLIP levels in endometrial carcinoma cells Oncogene 2008 27 18 2513 2524 17982483 10.1038/sj.onc.1210924

[B45] Unger GM Davis AT Slaton JW Ahmed K Protein kinase CK2 as regulator of cell survival: implications for cancer therapy Curr Cancer Drug Targets 2004 4 1 77 84 14965269 10.2174/1568009043481687

[B46] Meek DW Campbell LE Jardine LJ Knippschild U McKendrick L Milne DM Multi-site phosphorylation of p53 by protein kinases inducible by p53 and DNA damage Biochem Soc Trans 1997 25 2 416 419 9191128 10.1042/bst0250416

[B47] Kapoor M Lozano G Functional activation of p53 via phosphorylation following DNA damage by UV but not gamma radiation Proc Natl Acad Sci USA 1998 95 6 2834 2837 9501176 10.1073/pnas.95.6.2834 PMC19655

[B48] Landesman-Bollag E Channavajhala PL Cardiff RD Seldin DC p53 deficiency and misexpression of protein kinase CK2alpha collaborate in the development of thymic lymphomas in mice Oncogene 1998 16 23 2965 2974 9662328 10.1038/sj.onc.1201854

[B49] Uhle S Medalia O Waldron R Dumdey R Henklein P Bech-Otschir D Huang X Berse M Sperling J Schade R Protein kinase CK2 and protein kinase D are associated with the COP9 signalosome Embo J 2003 22 6 1302 1312 12628923 10.1093/emboj/cdg127 PMC151059

[B50] Cheong JW Min YH Eom JI Kim SJ Jeung HK Kim JS Inhibition of CK2{alpha} and PI3K/Akt synergistically induces apoptosis of CD34 + CD38- leukaemia cells while sparing haematopoietic stem cells Anticancer Res 2010 30 11 4625 4634 21115916

[B51] Dey A Tergaonkar V Lane DP Double-edged swords as cancer therapeutics: simultaneously targeting p53 and NF-kappaB pathways Nat Rev Drug Discov 2008 7 12 1031 1040 19043452 10.1038/nrd2759

[B52] Guzman ML Neering SJ Upchurch D Grimes B Howard DS Rizzieri DA Luger SM Jordan CT Nuclear factor-kappaB is constitutively activated in primitive human acute myelogenous leukemia cells Blood 2001 98 8 2301 2307 11588023 10.1182/blood.v98.8.2301

[B53] Chou TC Theoretical basis, experimental design, and computerized simulation of synergism and antagonism in drug combination studies Pharmacol Rev 2006 58 3 621 681 16968952 10.1124/pr.58.3.10

