# Supplementary material for: Inhibition of protein kinase CK2 with the clinical-grade small ATP-competitive compound CX-4945 or by RNA interference unveils its role in acute myeloid leukemia cell survival, p53-dependent apoptosis and daunorubicin-induced cytotoxicity
Source: J Hematol Oncol. 2013 Oct 12;6:78. doi: 10.1186/1756-8722-6-78 (PMC3852751; doi:10.1186/1756-8722-6-78)
Supplement: Additional file 1: Figure S1. — CK2 expression and activity in AML cell lines and normal mononuclear cells. (A) Real-time quantitative PCR analysis of CK2α mRNA expression in a panel of AML cell lines (K562, HL-60, NB4, ML2) and in normal peripheral blood (pb) cells. (B) Top: representative western blot analysis of CK2α protein expression in a panel of AML cell lines (K562, HL-60, NB4, ML2) and in normal peripheral blood (pb) or bone marrow (bm) cells; bottom: graph showing the corresponding densitometric analysis. [file 1756-8722-6-78-S1.pptx]

## Slide 1
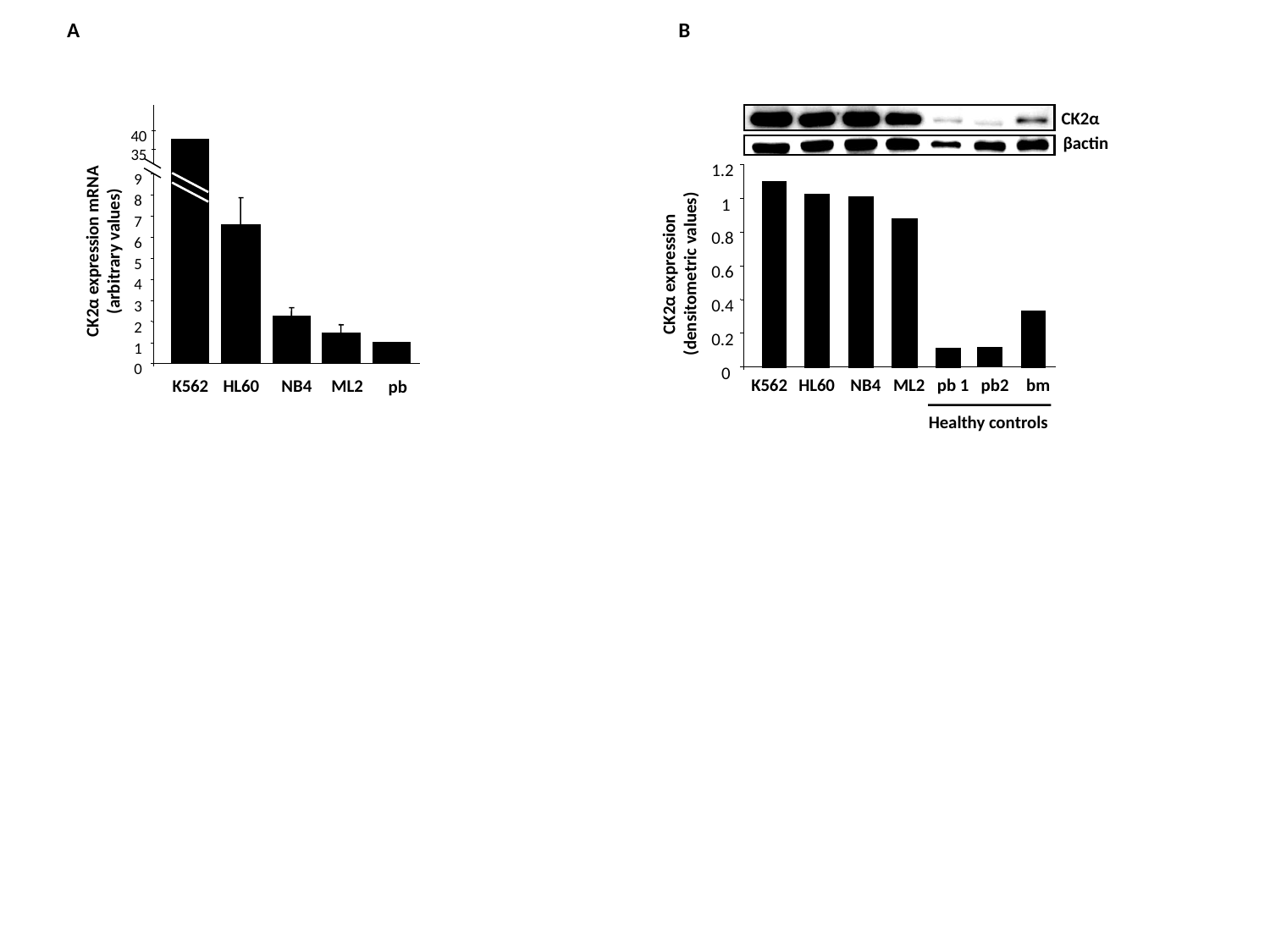

A
B
CK2α
βactin
1.2
1
0.8
0.6
0.4
0.2
0
CK2α expression
(densitometric values)
K562
HL60
NB4
ML2
pb 1
pb2
bm
Healthy controls
40
35
9
8
7
6
5
4
3
2
1
0
CK2α expression mRNA
(arbitrary values)
K562
HL60
NB4
ML2
pb
